# Psyche and soma: New insights into the connection

**DOI:** 10.4103/0019-5545.69238

**Published:** 2010-01

**Authors:** Rahul Kumar, Vikram K. Yeragani

**Affiliations:** M.S. Ramaiah Medical College and Hospital, Bangalore, India; 1 Departments of Psychiatry, University of Alberta, Edmonton, Alberta, Canada; Psychiatry and Behavioral Neurosciences, Wayne State University School of Medicine, Detroit, MI, USA; Biotechnology, Acharya Nagarjuna University, AP, India

**Keywords:** Mind-body connection, psyche and soma, Psychoneuroimmunology

## Abstract

The interaction of Psyche and Soma are well known and this interaction happens through a complex network of feedback, medication, and modulation among the central and autonomic nervous systems, the endocrine system, the immune system, and the stress system. These systems, which were previously considered pristinely independent, in fact, interact at myriad levels. Psychoneuroimmunology (PNI) is an emerging discipline that focuses on various interactions among these body systems and provides the underpinnings of a scientific explanation for what is commonly referred to as the mind-body connection. This article reviews the relevant literature with an emphasis on Indian research.

## INTRODUCTION

Integral physiology has to do with the synthesis of conventional physiology and how our individual psyches (i.e., mind, emotions, and spirituality) interact with the world around us, to induce positive or detrimental changes in our bodies. In a broader sense, the concept applies to the health of society as a whole. In the past two decades, biomedical research has changed our understanding of body systems. It has now come to light that there is a complex network of feedback, mediation, and modulation among the central and autonomic nervous systems, the endocrine system, the immune system, and the stress system. These systems, which were previously considered pristinely independent, in fact, interact at myriad levels. Psychoneuroimmunology (PNI) is an emerging discipline that focuses on various interactions among these body systems and provides the underpinnings of a scientific explanation for what is commonly referred to as the mind-body connection. One should not construe here that all the phenomena are finally mediated only through immune mechanisms.

## EMERGENCE OF PNI

In 1964, George Freeman Solomon wrote “Emotions, immunity, and disease: A speculative theoretical integration.” In this article, Solomon first used the term ‘*psychoimmunology*’ and introduced the concept of a medical link between our emotions and immune systems.[1] In 1975, Ader expanded on Solomon’s work and coined the term ‘*PNI*’. During that same year, Ader and his colleagues published the startling results of their research on the conditioned immune response in a rat population.[2] The rats in the experimental group were injected with cyclophosphamide (an immunosuppressive agent), while simultaneously being given drinking water flavored with saccharin. The rats were later given only the saccharin-flavored water but no cyclophosphamide. To the researchers’ surprise, the rats continued to evidence immunosuppression. This was the first documented example of Pavlovian conditioning of the immune response. In Ader’s groundbreaking research, he used a pharmaceutical agent to induce the conditioned immune response. Subsequent studies have expanded on the theory to include investigations of conditioning stimuli that are neither physical nor chemical, but are instead cognitive (e.g, perceptions, thoughts, or emotional states). What has been discovered is that these cognitive stimuli can just as easily mediate changes in the immune system. Two noteworthy examples often quoted in the context of PNI are mentioned; one is that lymphocyte activity in men diminishes immediately after the death of a spouse from breast cancer,[3] and second, a study of 75 medical students showed a significant reduction in natural killer-cell activity during the final examinations as compared to the previous month.[4]

Twenty years later, *Lancet* published a study by Ader and Cohen that concludes with the following statement: “The association between stressful life experiences and changes in immune function do not establish a causal link between stress, immune function, and disease. This chain of events has not been definitively established”.[5] Thus, the unifying link remained elusive for a large part of the late twentieth century. Only recently have major breakthroughs occurred that have revolutionized our understanding of PNI. In this article, we will make an attempt to demonstrate the integration among body systems and also the causal link can now be established between these systems based on the available knowledge.

## CONDITIONED IMMUNE RESPONSE

The key phenomenon that is the basis for the discipline of PNI is the so called ‘Conditioned Immune Response’. Research elucidating the interactions between the nervous and immune systems began with studies on conditioned responses of the immune system. As mentioned earlier, in 1975, Robert Ader and his colleagues published their research on the conditioned immune response in a rat population. Similarly, over a hundred years ago, Sir William Osler, the notable physician from John Hopkins, described a patient having an asthma attack after smelling an artificial rose. Although the effects of such conditioning were experientially familiar to physicians, Ader’s experiment was the first scientific proof of Pavlovian conditioning of the immune response. Ader’s research opened the way to a plethora of studies that illustrate the conditioning of immune suppression, and to some that define immune enhancement as well. Conditioned immune enhancement, like suppression, has now been illustrated with the use of the same chemical, cyclophosphamide, as well as by a variety of other stimuli, including taste and smell.[6] However, much of the earlier research on conditioning involved studies of immune suppression. Many of these studies showed that an aversive stimulus can induce glucocorticoid elevation and immune suppression. It is clear that the hypothalamic-pituitary-adrenal (HPA) axis is a predominant pathway for neuromodulation of the immune system. Ader’s work also revealed that antibodies can increase by simply using an antigen as the unconditioned stimulus, postulating that it is the interaction between the immune and neuroendocrine systems that mediates the conditioned response.[7] All of this research suggests that behavior itself is the regulator of immune function.[8]

In this context, we are not ignoring the effect of genetic inheritance, but given that the genes contribute to a particular extent, it is the individual’s environment that may decide the final manifestation of an illness. As we mentioned the HPA axis, it is noteworthy to give credit to all the modern work linking the paraventricular nucleus to the production of the corticotrophin-releasing hormone (CRH), which eventually results in cortisol-mediated stress reactions in the body, which can include several different illnesses including cardiovascular (CV) illnesses and sudden death. Our previous and current studies have been examining how anxiety and depression may finally affect the cardiovascular system, leading to an increase in mortality and morbidity. Recent studies have again shown a link between glutamate receptors and some of these mechanisms.

## THE CONNECTING LINK

Many neurotransmitters and their receptors, previously thought to be located only in the brain, have been found in the immune system.[8] Conversely, accumulated research shows that any immune function can occur in the brain. When the central nervous system (CNS) receives cognitive stimuli that are relevant to the immune system, it conveys that information by hormonal pathways to receptors on immune cells, causing immunological changes. For example, g-aminobutyric acid (GABA) receptors (GABA being the primary inhibitory neurotransmitter) and benzodiazepine receptors (benzodiazepines being powerful anti-anxiety molecules), typically thought of as being housed in the brain, have been discovered on immune cells and actually modulate the actions of the immune system.[9] This is the physical basis for the mind’s impact on the development of disease — a primary example of the mind-body connection.

The nervous system communicates with the immune system via sympathetic fibres coming from and going to the brain. The fibres innervate the primary (i.e, bone-marrow, thymus) and secondary (i.e, spleen, lymph nodes) immune organs including the noradrenergic, cholinergic, and peptidergic nerve fibers.[10] Neurotransmitters must be typically activated by the immune system before passing on their message. Therefore, how does the brain receive and respond to chemical and electrical information from the immune system?

The CNS is capable of modulating the immune system from within the CNS itself (e.g, the microglia have phagocytic functions in the brain). However, modulation predominantly occurs via peripheral immune stimuli affecting the autonomic nervous system (ANS). The information received involves messages with regard to the general type and level of intensity of the intruder, and not information about the specific antigen. In other words, the immune system alone detects an antigen, virus, or bacteria. It then lets the central and peripheral nervous systems in on the news, by way of its own mediators as well as via neuroendocrine mediators. The immune system’s activation of the CNS most likely involves the older brain structures, such as the limbic system, and follows discrete neuronal pathways.[11] Interestingly, the immune stimulus (e.g., virus, bacteria, etc.) must reach an, as yet, undetermined but apparent threshold before it is capable of activating the CNS. The CNS can then generate the neuroendocrine peripheral effects. There is, in fact, an interactional and functional relationship between the two systems. For example, when secreted from the sympathetic nerves, epinephrine and norepinephrine generally *suppress* the immune system, but both have distinct immune *enhancing* effects in the CNS, potentiated by the immune system’s own cytokines, interleukin (IL)-1 and -2.[12] Based on these findings, researchers have designated the immune system a *sensory organ* for its ability to obtain, process, and then dispatch information to the CNS. One of the greatest examples of the interdependency of the nervous and immune systems came out of the pioneering work that began in the late 1970s, which was performed by Hugo Besedovsky and his colleagues in Germany. They determined that the neuronal firing rates increased in the hypothalamus during the peak antibody response to an immunization, with a corresponding decrease in norepinephrine content of the hypothalamus. Norepinephrine also showed a time-dependent decrease in the spleens of mice following immunization, as well as after an antigen challenge.[13–16] Ten years later, a pattern of increased firing rate corresponding to antibody production was ascertained by another investigator as well.[17] Any alteration in neuroendocrine factors, whether local or systemic, can markedly alter the immune activity. Given the mobile nature of immune cells, messages can reach the immune system through nerves in the vicinity of the target immune cells or via circulation (i.e., local or systemic influences). The first evidence that immune/brain communication causes a peripheral response was the observation that glucocorticoid levels increase when the HPA axis is activated. This systemic change results in immune system adjustments. Likewise, local synthesis and secretion of neuropeptides by immune cells are important for subtler adjustments in the maintenance of immune homeostasis. Research has eventually focused on the precise modulating activities of the neuropeptides as they affect the immune cell function of the immune cells on the neuroendocrine tissue and organs. One must bear in mind that the body systems are sharing receptors for multiple possible combinations of immune, endocrine, stress, and/or nervous system factors that can be elaborated either within or between one another. Cytokines are the immune system’s own mediators and are capable of modulating the immune system in a localized manner. For example, IL-1 stimulates itself as well as the tumor necrosis factor (TNF), IL-2, and IL-6, which results in immune modulation.[18] In addition, cytokines are the principal mediators of communication between the immune and neuroendocrine systems, which also result in immune system modulation, particularly with regard to inflammation and infection. The immune system has receptors for foreign stimuli, such as antigen, virus, or bacteria, which, as mentioned, the CNS is incapable of recognizing on its own. However, the immune system can communicate the presence of such stimuli through cytokine immunological messengers.[19]

On recognition of the cytokine by the CNS, the information is converted to neuroendocrine signals, resulting in chemical messages being sent back to the immune system, with ensuing physiological changes. By and large, the cytokines (and their receptors) that are found in the nervous system are localized to the brain. Although most research has been performed on rodents, TNF and interferon γ (INF γ) have been found in human brain tissue and IL-1 in the tissues of human hypothalamus, thyroid, and ovary as well. A detailed analysis shows that different cytokines have discrete portions of the brain that they are capable of stimulating: Dopamine in the striatum, prefrontal cortex, and hippocampus; serotonin predominantly in the hippocampus; and tryptophan accumulating in a more diffuse fashion in the CNS.[20,21] The effect of having cytokines localized in the brain was that they were capable of influencing neuroendocrine production. Among the first cytokines found to have hormonal function were INF, which increased glucocorticoid production, and IL-1, which increased the hypothalamic secretion of the corticotropin-releasing hormone (CRH).

However, now we know that cytokines are responsible for numerous neuroendocrine alterations. The activated immune system sends both humoral and neural messages to the brain that there is some type of intruder (antigen, virus, or bacteria) present in the body. On recognition of the cytokine by the CNS, the brain converts the information to neuroendocrine signals, resulting in chemical messages being sent back to the immune system. The CNS response to the cytokine message either affects distinct neuroendocrine functions that are under the control of the CNS (e.g., stimulating the HPA axis), or it promotes the behavioral properties of peripheral cytokines (e.g., fever). The hypothalamus, hippocampus, and the pituitary of the CNS, as well as, the sympathetic nerve terminals of the peripheral nervous system are the primary sites at which communication occurs.[22] Another route for cytokine modulation in the CNS is via the immune cells themselves. Activated immune cells are capable of permeating the blood-brain barrier and secreting cytokine mediators. This interaction is distinct from the cytokines independently traveling to the CNS. Studies show that these brain-born cytokines can influence peripheral neuroendocrine functions and influence behavioral effects, particularly those associated with the hypothalamus and hippocampus. These actions probably help maintain homeostasis, by modulating the interaction of the systems during antigen challenge. Moreover, a fascinating research shows that IL-1, IL-2, IL-6, TNF α, and INF γ, all cause pituitary-like hormones to be secreted by immune cells in a localized autocrine- and paracrine-type manner. This news is astounding, and the implications for the modulation and integration of systems are profound. These lymphocyte-derived, pituitary-like hormones actually modulate subtle adjustments in pituitary hormone secretions. For example, IL-1 regulates anterior pituitary cell growth, while IL-2 and IL-6 inhibit normal growth yet encourage tumor growth.[23] As for the other aspects of immune-neuroendocrine bidirectional communication, we see that the cytokines play an enormously important role in system homeostasis during immune challenges.

### Integration: The potential for harmony

Chemical and electrical transmitters, once thought to have limited and discrete functions, are found to have a significant impact on one another, often interchanging functional roles. Although studies bringing to light specifics such as the fact that lymphocytes have receptors and secrete neuropeptides are of enormous significance to medical science, and the intricacy in systems interaction that will be revealed in the coming decade will be far more astounding. Now that scientists have discovered the functional modulators that have the most dynamic influence on the body, increasingly subtler ones are being detected. The HPA axis is connected to a memory system for stress and trauma. We can now begin to speculate that the immune system also has a memory beyond that which is specific to the antigen memory. The same sites (e.g., the hippocampus and hypothalamus) that are recognized as crucial for memory functions of stress are also fundamentally important in the immune-neuroendocrine bidirectional communication pathway. Both these sites are important transfer stations for cytokines — the all-important interceding messengers. The ubiquitous and intricate array of electrical and chemical routes of communication that are already known to make up immune response are a compelling indication that there could be a memory for the emotional or behavioral components of an illness. What are some of the practical implications of understanding that our bodies are integrated networks? We know that illness and psychosocial factors, such as stress, bereavement, or divorce, can change or deplete immune performance and alter neuroendocrine function. The impact of these events on one’s health is understood more fully from the perspective of systems integration. Our bodies have the capacity to function with separate, yet fully interactive parts maintaining homeostasis. There is a harmony, whose sum is greater than the parts — in other words, there is integral physiology.

### Mind body interactions and their influence on the pathophysiology of disease

We toil with the understanding that cells of the immune system have receptors for neurotransmitters, neuropeptides, and hormones, and we embrace the notion that primary and secondary lymphoid tissues are innervated by the sympathetic nervous system. We have a solid working knowledge of how stressors activate the hypothalamic-pituitary-adrenal axis and the sympathetic nervous system, and we also have an appreciable grasp of how products of these systems affect molecular and cellular changes in the immune system.

As far back as the early twentieth century, investigators who were undertaking hypothesis-driven research in psycho somatic medicine demonstrated the importance of mind-body interactions in the pathophysiology of disease. In the 1930s Walter Cannon was one of the first to note that fear and stress contributed to the development of physical symptoms akin to heart disease.[24] Subsequently, it was noted that multiple psychological risk factors were present in a large population of patients with heart disease. These could be chronic personality traits, such as hostility; there could be episodic factors, which could include depression and exhaustion; or risk factors that could manifest in the form of something like anger, which was classified as an acute psychological trigger. As the years passed, since Cannon’s observations, the results of an increasing number of studies suggested that the immune system played an important role in the relationship between these psychological risk factors and future coronary syndromes.

Psychological factors have also been implicated in the onset and exacerbation of various skin disorders. Dysregulation of the hypothalamic-pituitary-adrenal axis and sympathetic-adrenomedullary system are proposed to aggravate the allergic inflammatory process in atopic dermatitis.[25]

While the influence of stress on the development of cardiovascular and inflammatory diseases was among the first areas in psychoneuroimmunology to receive attention, the field broadened significantly, as the role of inflammation in numerous central and peripheral processes became evident. The adipose tissue is now recognized as a dynamic organ with important roles in modulating satiety, reproduction, immunity, and metabolism. More importantly, it has been reported that the obese exhibit increased markers of inflammation including macrophage infiltration. Studies on the relationship between inflammation and obesity may provide information on the pathologies associated with obesity.[26] PNI also has a pivotal role in host defenses. Pathogen recognition has been a major focus of immunological research for the past decade. Studies on innate resistance and the mechanism by which a mammal initially recognizes a viral or bacterial challenge have provided an insight into basic host resistance. The long-held dogma of ‘self versus non-self’ discrimination as the capstone of immune response has been modified to include the concept of ‘danger signals’.[27]

Danger signals are elicited in damaged tissues in response to insult/injury, and they provide information through primitive receptor-ligand interactions, as part of an innate resistance. Exposure to mental or physical stressors stimulates a cascade of behavioral and physiological responses that are directed at improving an organism’s chance of survival. At the cellular level, the induction of heat shock proteins — specifically heat-shock protein 72 (Hsp72) — may be a previously unrecognized feature of an acute stress response. Hsp72 may function as an endogenous ‘danger signal,’ leading to the facilitation of immune responses during times of acute stress.[28] Various infectious diseases commonly befall the average person, and diseases caused by bacteria and viruses continue to be a significant cause of morbidity and mortality worldwide. Psychoneuroimmune interactions are also critical factors that influence microbial pathogenesis and individual susceptibility to infection. A large body of research has linked psychosocial factors to variations in HIV disease progression. However, the biological processes mediating those effects remain poorly understood. The biological signaling pathways that convey the effects of the psychosocial factors to the cellular and molecular processes involved in HIV pathogenesis have been better understood in recent years.[29] A latent viral infection (herpes simplex virus, HSV) that is associated with stress-induced reactivation has also helped the scientists to study the PNI aspects of stress inducing such a phenomenon.[30]

**Figure 1:**
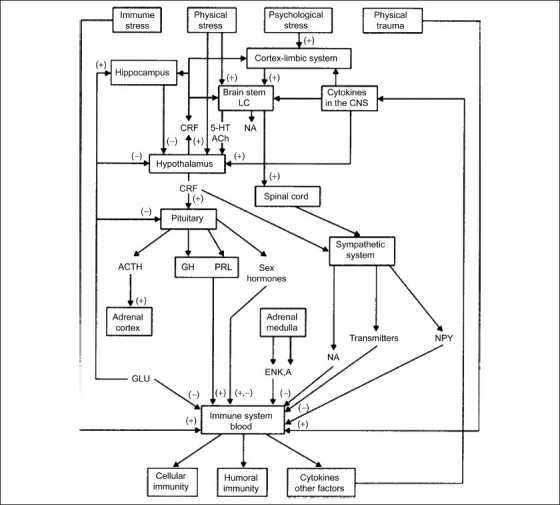
Neuro-anatomical/physiological basis of psychosomatic responses

Stress-induced neuroendocrine interactions are also known to influence the immune system during a viral infection, which may possibly lead to an autoimmune disease. A Theiler’s virus infection of the central nervous system model is being used for the study of multiple sclerosis (MS), and the basic pathology of the disease and the present evidence do support the role of viral infection in the etiology of MS. The role of stress on the development of MS has also been proved, paying particular attention to the anti-viral immune responses in the development of the disease.[31] Based on the existing body of evidence, an attractive model of the Psycho-Neuro-Immune axis has been proposed and is presented here [Figure 1].

### The Indian scenario

Ultimately all the hypotheses should be proven in the systematic, basic, and clinical research, not only to identify the risk and mechanisms that mediate an illness, but also to know how various treatments can be beneficial. For example, all our ongoing work on the risk factors of cardiac mortality and morbidity, associated with anxiety and depression, is a miniature attempt to understand one aspect of an elaborate phenomenon.[32] Our recent work on how physical exercise may affect the repolarization lability in ventricular myocardium is one such example.[33] In this context some other works on the effects of the brain on the mediation of cardiac repolarization is exciting, as it links the medial prefrontal cortex, the QT interval. Likewise, it will be very fruitful to understand the mechanisms of many modalities of treatments, where the psychological, spiritual, and pharmacological means may yield valuable information in future, delineating the sympathovagal balance in the system.

In the Indian context, an association between the psyche and well being has been referred to in the ancient Epics such as the Ramayana, Mahabharata, and the Bhagwat Geeta. In the modern era, the first reference to this topic was published in Psychosomatic Medicine in 1946.[34] The author reviewed the charts of 1266 consecutive patients treated by him in a year on a General Medical Section of an Army hospital in Assam, India. Nine hundred and twenty-eight patients (73.3 percent) had organic, somatic diseases; 104 (8.2 percent) had full-blown psychoneurosis; 234 (18.5 percent) had a combination of the two: Psychosomatic illnesses. In other words, one out of every four general medical patients had a large emotional component to his illness. Of late, the impact of Psychoneuroimmunology has also been addressed in relation to tuberculosis[35] and the field of Medical Oncology.[36] However, an original research on the topic is lacking and further studies are needed in the Indian perspective, as the dynamics and family support structure is different in India when compared to the West.

The authors have carried out a study on the Intensive Care Patients, where the outcome was compared to the coping skills of the patients (unpublished data). The coping skills were assessed using the Bell’s inventory and the style-questionnaire and were compared with the final outcome. The cohort of patients studied were patients with sepsis. The assessment of neuroimmune functions was done by estimating the Tumor Necrosis Factor α, Interleukin 1, and Interleukin 6 levels. A direct correlation was noted between optimism and good coping skills to a favorable outcome. It was also noted that the immune response was more streamlined in individuals having good coping skills.

In an interesting and thought provoking editorial, Sathyanarayana *et al*. have addressed the issue of the control of the mind over body.[37] They mention “The biochemistry of our body stems from our awareness.[38] Belief-reinforced awareness becomes our biochemistry. Each and every tiny cell in our body is perfectly and absolutely *aware* of our thoughts, feelings, and of course, our beliefs. There is a beautiful saying ‘Nobody grows old. When people stop growing, they become old’. If you believe you are fragile, the biochemistry of your body unquestionably obeys and manifests it. If you believe you are tough (irrespective of your weight and bone density!), your body undeniably mirrors it. When you believe you are depressed (more precisely, when you become consciously aware of your ‘Being depressed’), you stamp the raw data received through your sense organs, with a judgment — that is your personal view — and physically become the ‘interpretation’ as you internalize it. A classic example is ‘Psychosocial dwarfism’, wherein, children who feel and *believe* that they are unloved, translate the perceived lack of love into depleted levels of the growth hormone, in contrast to the strongly held view that the growth hormone is released according to a preprogrammed schedule coded into the individual’s genes!”

## CONCLUSION

It is not at all an exaggeration if we state that there is much more to be learnt in spite of all the above advances. Hopefully, we may identify the exact neuronal pathways of the psychological effects and their mediation on the soma. However, we already see a lot of fruits resulting from this.
